# Changes in Prefrontal-Limbic Function in Major Depression after 15 Months of Long-Term Psychotherapy

**DOI:** 10.1371/journal.pone.0033745

**Published:** 2012-03-28

**Authors:** Anna Buchheim, Roberto Viviani, Henrik Kessler, Horst Kächele, Manfred Cierpka, Gerhard Roth, Carol George, Otto F. Kernberg, Georg Bruns, Svenja Taubner

**Affiliations:** 1 Institute of Psychology, University of Innsbruck, Innsbruck, Austria; 2 Department of Psychiatry and Psychotherapy III, University of Ulm, Ulm, Germany; 3 Hanse Institute for Advanced Study, Delmenhorst, Germany; 4 Department of Psychosomatic Medicine and Psychotherapy, University of Ulm, Ulm, Germany; 5 Department of Psychiatry, University of Bonn, Bonn, Germany; 6 International Psychoanalytic University, Berlin, Germany; 7 Institute of Psychosomatic Cooperation Research and Family Therapy, University of Heidelberg, Heidelberg, Germany; 8 Brain Research Institute, University of Bremen, Bremen, Germany; 9 Department of Psychology, Mills College, Oakland, California, United States of America; 10 Weill Medical College, New York, New York, United States of America; 11 University of Bremen, Bremen, Germany; 12 Department of Psychology, University of Kassel, Kassel, Germany; University of Missouri-Kansas City, United States of America

## Abstract

Neuroimaging studies of depression have demonstrated treatment-specific changes involving the limbic system and regulatory regions in the prefrontal cortex. While these studies have examined the effect of short-term, interpersonal or cognitive-behavioural psychotherapy, the effect of long-term, psychodynamic intervention has never been assessed. Here, we investigated recurrently depressed (*DSM-IV*) unmedicated outpatients (*N* = 16) and control participants matched for sex, age, and education (*N* = 17) before and after 15 months of psychodynamic psychotherapy. Participants were scanned at two time points, during which presentations of attachment-related scenes with neutral descriptions alternated with descriptions containing personal core sentences previously extracted from an attachment interview. Outcome measure was the interaction of the signal difference between personal and neutral presentations with group and time, and its association with symptom improvement during therapy. Signal associated with processing personalized attachment material varied in patients from baseline to endpoint, but not in healthy controls. Patients showed a higher activation in the left anterior hippocampus/amygdala, subgenual cingulate, and medial prefrontal cortex before treatment and a reduction in these areas after 15 months. This reduction was associated with improvement in depressiveness specifically, and in the medial prefrontal cortex with symptom improvement more generally. This is the first study documenting neurobiological changes in circuits implicated in emotional reactivity and control after long-term psychodynamic psychotherapy.

## Introduction

Neuroimaging approaches have been used to investigate neurobiological changes in depressive patients in studies that address remission after psychotherapy or antidepressant medication [1]–[2]. The majority of these studies focused on the effect of treatment on brain metabolism or perfusion at rest. A more recent series of functional neuroimaging (fMRI) studies has turned to the use of experimental tasks to examine processes that may be more directly involved in emotional appraisal and control processes [3]–[6]. To date, studies examining the functional neuroanatomy of psychotherapy in depressed patients have applied interpersonal therapy or cognitive behavioural therapy [7]–[8]. The present research reports on the first fMRI study with recurrently depressed patients treated with psychodynamic psychotherapy. Long-term psychodynamic psychotherapy has been shown to be associated with larger improvement in these difficult forms of depression than shorter treatments [9]–[10].

The interest aroused by neuroimaging studies of treatment of depression is in part due to their potential importance in shedding light on the mechanism of therapy. There is considerable evidence for increased activation in limbic areas in depression, especially the amygdala, under exposure to emotional stimuli [5]–[8], [11]–[12]. This limbic activation may be related to an increased reactivity of depressed patients to emotional stimuli of negative valence [13]. It has been proposed that antidepressant medication acts directly on this abnormal reactivity [1], since amygdalar activation normalizes in medicated remission [5]–[6]. However, changes associated with depression have also been reported for prefrontal regions during the execution of cognitive tasks [14]. Because of the regulatory nature of many processes mapped onto the prefrontal cortex, these changes may refer to mechanisms involved in emotional regulation during the acute depressive episode, or reflect deficits in cognitive control [15]–[16]. Different interpretations of signal changes in the prefrontal cortex in depression or after therapy may be given [17]. Given that emotion regulation may either have adverse or protective roles in mental health, depending on the mechanism through which it operates [18]–[19], it may be important to determine not only if, but also how emotions are regulated [20].

Psychodynamic approaches propose that depression, besides its biological and social underpinnings, may be meaningfully conceived as a specific organization of an individual's conscious or unconscious beliefs and feelings. The resulting mental operations constitute defensive strategies that aim at avoiding negative feelings arising out of conflict in order to maximize a subjective sense of safety [21]. The objective of long-term psychodynamic psychotherapy is a stable modification of these strategies that allows the patient to work through, achieve insight, and reappraise experiences that are related to depressive pathology. Therefore, in our study we would expect neural patterns of appraisal of sensitive material to change during therapy and move from emotion regulation styles characterized by unfavourable strategies to more integrated acceptance and awareness. The identification of these neural patterns was the general aim of our study.

To date, only one fMRI study has investigated the effect of psychotherapy (cognitive behavioural therapy) on major depression using a standardized experimental task (Fu et al., [22]). In this study, participants were exposed to faces portraying different degrees of sadness. This study detected changes in the amygdala-anterior hippocampus and posterior cingulate-precuneus regions as well as the superior frontal gyrus, suggesting that areas associated in previous studies with increased reactivity to emotional stimuli and mechanisms of control are also relevant for psychotherapy effects.

In the present study, patients with recurrent unipolar depression underwent functional neuroimaging scans at the beginning of treatment and after 15 months, during which they were treated with psychodynamic psychotherapy by experienced therapists. A matched healthy control group without psychotherapy was scanned at the same time points. The stimulus materials were attachment-related pictures from the Adult Attachment Projective Picture System (AAP [23]), a measure that has been shown to be valid for use in an fMRI environment [24]–[25]. These pictures are designed to elicit mental engagement with attachment-related experiences such as separation, illness, danger, and loss. The fundamental ability to form attachment is indispensable for human social relationships. Since Bowlby's seminal contributions [26], attachment and separation have become essential theoretical components of developmental psychology, and psychodynamic theory [27]. One key feature of interpersonal problems in depressed patients is their feelings of helplessness and fear of losing the love of a significant other [28]. Internal representations of the self as unlovable and of attachment figures as unloving are a central dimension of Beck's cognitive triad of depression [29].

To increase the capacity of the signal to elicit a response related to the emotional processes of each individual, material was here prepared using personalized content [11], [30]–[32] derived from AAP interviews with each participant. In the personally relevant condition the AAP attachment scenes were accompanied by individually tailored descriptions containing core sentences from the patient's own narrative previously elicited by each picture (see Material and methods for details). The same series of attachment scenes accompanied by a standard factual, non-emotional description for all participants was used as control condition.

In addition to the detection of a neural signature of treatment, we were interested to see the extent in which the pattern of change corresponded or differed from the one described by Fu et al. [22]. On the basis of this study, we formulated specific hypotheses relative to the general aim of the study of characterizing changes in neural patterns of appraisal and emotion regulation occurring after therapy. Our first hypothesis was the normalization of emotional reactivity indexed by changes in the amygdala-anterior hippocampus region, as found by Fu et al. [22]. Our second hypothesis was the existence of changes in prefrontal areas detected in previous studies [22], [31], [33] as possible markers of the specific effect of psychodynamic psychotherapy on styles of emotion regulation [1], [8].

The present design differed from that of previous studies in several further respects. As mentioned before, neuroimaging studies have examined the effects of short time psychotherapy (e.g. 12–20 weeks), applying cognitive-behavioural or interpersonal therapy [7]–[8]. We examined depressed patients with a history of several previous depressive episodes during psychodynamic treatment providing a longer observation window (15 months of therapy) than in previous studies.

The present investigation therefore complements the existing emerging picture of changes associated with the therapy of depression emerging from neuroimaging studies. In meta-analyses of neuroimaging studies of the effect of psychopharmacological intervention [34], decreased activation following treatment was reported in the anterior hippocampus and parahippocampal cortex, in the subgenual and pregenual cingulus, in the insula, and the putamen. In the prefrontal cortex, decreased activation was reported in the middle and superior left frontal gyrus. The frontal lobes, however, were also the seat of several meta-analytic foci of increased activation following treatment (in the middle frontal gyri bilaterally and the dorsal anterior cingulate cortex). Further increased activation was found in the posterior cingulate cortex and in temporo-parietal regions on both sides. With the exception of Fu et al. [22], neuroimaging studies of psychotherapy of depression have examined only resting state metabolism (for reviews, see [7]–[8], [35]). Here, therapy outcomes were associated primarily with changes in the prefrontal cortex (dorsolateral, ventrolateral, and medial) [36]–[38], but the direction of changes was not entirely consistent. Some studies reported reduced rest metabolism or perfusion that normalized at endpoint [37]–[38]. In other studies, however, this finding was reversed [36]. When both psychotherapy and pharmacotherapy were compared [37], there was limited overlap between changes found in these two therapy modalities. While all these studies appear to be broadly compatible with the involvement of limbic and prefrontal areas, more studies of task-related activation are needed to provide a comprehensive picture of changes associated with remission.

## Materials and Methods

The study protocol was approved by the ethical committee of the University of Ulm and was in compliance with national legislation, the principles expressed in the Declaration of Helsinki, and the Code of Ethical Principles for Medical Research Involving Human Subjects of the World Medical Association. All participants gave written informed consent.

### Participants

Patients were recruited from the outpatient departments of two psychoanalytic institutes in Bremen, Germany, and diagnosed by two trained clinicians using the Structured Clinical Interview for DMS-IV Diagnosis (SCID, German version, [39]). Eight patients were diagnosed with double depression (dysthymia and former major depression episodes); in the remaining patients, degree of depression was severe. Ten patients had a comorbid anxiety disorder. Patients reported 3–10 previous depressive episodes (mean 6 episodes, standard deviation 3.4). Age at first occurrence of depression was between 8 and 37 years (mean 20 years, standard deviation 9.2). All patients reported previous unsuccessful psychopharmacological and/or psychotherapeutic treatment (none of which was psychodynamic). Exclusion criteria were other psychiatric conditions as main diagnosis, substance abuse, significant medical or neurological conditions (including medical causes of depression) and psychotropic medication. Inclusion criteria were a long history of recurrent major depression as an appropriate indication for psychodynamic treatment. Non-depressed controls were recruited from the community, matched for age, sex and education; control participants had no history of previous depressive episodes or other psychiatric conditions (SCID). Ethical considerations precluded the recruitment of a depressed group left untreated for the duration of the study. All participants were right-handed.

Depression severity and general psychological symptoms were assessed using the Beck Depression Inventory (BDI, [40], German version [41]) and the Global Severity Index (GSI) from the revised Symptom Check List (SCL-90-R [42], German version [43]). Of the initial sample of 38 participants, 5 dropped out of the study (3 controls and 2 patients). The final sample of 33 consisted of 16 patients and 17 controls (7 males, mean age 38.9 years, standard devaition 12.4, range 20–64). Patients and controls in the final sample did not differ in age (logistic multiple regression, *z* = 0.41, *p* = 0.68), sex (*z* = 0.49, *p* = 0.62), and education (*z* = 0.56, *p* = 0.57).

### Treatment

Patients were treated with long-term psychodynamic psychotherapy by 16 formally trained psychoanalysts (mean years of experience 22.4, standard deviation 7.9). Training as a psychodynamic psychotherapist is certified by the German state and regulated by laws specifying the hours of theory, psychotherapy under supervision, and self-awareness training. Patients underwent 2 to 4 hours of therapy weekly (2 patients had 4 hours, 7 had 3 hours, and 7 had 2 hours per week). At the end of the study period of 15 months, patients had received from 90 to 210 hours of psychotherapy (mean 129 hours, standard deviation 37). Psychotherapy formally qualified as psychodynamic on the basis of two criteria. The first was observance of the “couch setting” in which the patient talks while lying on a couch with no visual contact with the therapist. The second were the core features of psychodynamic thinking and therapeutic technique, which include interventions focusing on the interpretation of the patient's unconscious conflicts as they emerge in the transference relationship [21], and the focus on affect as it emerges in relationships, in attempts to avoid distressing thoughts, in memory of past events, and in recurring patterns of interactions [44]. Adherence to these principles was assured by a regular case-discussion group led by one co-author (GB) in which all participating therapists presented their patients and interventions. Participants to this group had no access to the material produced by patients during the AAP interview and its results used to create personalized stimuli in the scanner.

All patients were free of psychotropic medication throughout the entire 15 months of the study by their own choice. After the end of the study, therapy continued for varying periods of time, for total therapy lengths ranging between 24 and 48 months in accordance to individual therapeutic contracts, course of treatment, and health insurance allowance.

### Experimental design

**Figure 1:**
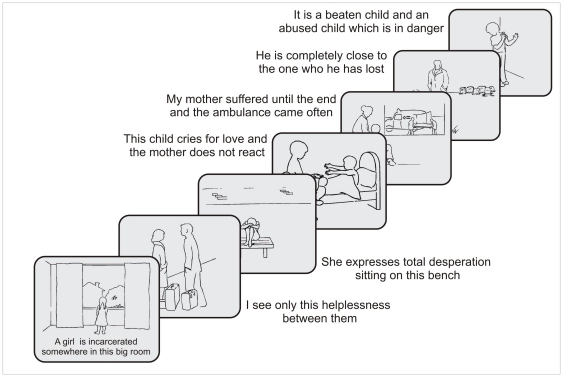
Stimuli. Prototypical presentation with personally relevant sentences from the AAP Picture System.

Stimuli were derived from the Adult Attachment Projective Picture System (AAP, [23]), an established and validated interview to assess attachment patterns. The AAP consists of 7 picture stimuli, designed to activate the attachment system [25]. Two to four weeks prior to the fMRI experiment, one trained judge (ST) conducted a standard AAP interview. Administration involves asking participants in a semi-structured format to describe the scene in the picture, including what characters are thinking or feeling, and what they think might happen next. Three core sentences that represented the attachment pattern of the participants were extracted from the audiotaped responses to each AAP picture stimulus by two independent certified judges (e.g., “A girl is incarcerated in that big room”, “My mother suffered until the end and the ambulance came often”). These sentences were paired to the respective picture to constitute the “personally relevant” trials tailored to each participant (Figure 1). The same pictures, paired to sentences describing only the environment of the depicted situation (e. g. “There is a window with curtains on the left and right”, “There is a bed with a big blanket”) constituted the “neutral” trials, and were identical for all participants. Each trial consisted of the presentation of the picture-sentence pair for 5 seconds, followed by a fixation cross between 7.5 and 12.5 seconds. Participants were instructed to mentally engage with the attachment scene in the picture and its textual description.

Stimuli were presented in two blocks of six trials each. Each block contained three different sets of personally relevant sentences and neutral sentences for each picture stimulus. Trials alternated between the personally relevant and neutral in groups of seven AAP picture stimuli. In total, there were 84 trials resulting in a scanning time of about 21 min. After scanning, participants filled out a questionnaire that asked to rate on a 7-point Likert scale the degree of emotional arousal and relevance of the personally related sentences (emotional arousal is here a construct referring to the degree to which participants rated personal sentences emotionally involving, not the degree of depressive symptoms associated with them). There were no significant differences in the ratings of patients and controls (*t*_31_ = 0.31, *p* = 0.38), thus suggesting that differences in neural activation levels between patients and controls were not driven by higher emotional valence of the stimuli.

### Image acquisition

MRI data were obtained with a 3-T Magnetom Allegra head scanner (Siemens, Erlangen, Germany), equipped with a standard quadrature head coil. To reduce anxiety levels, anatomical images were acquired first (3D high resolution T1-weighted volume, MPRAGE-sequence; TR/TE/TI = 2300/4.38/900 ms, flip angle = 8°, FOV = 256×256×176 mm, isotropic voxel size 1 mm, total acquisition time 7.5 min). A total of 508 EPI T_2_*-weighted whole brain volumes were acquired (TR/TE = 2500/30 ms, flip angle 90°, FOV 192 mm, matrix 64×64, voxel size 3×3 mm, slice thickness 3 mm, 44 slices, interleaved acquisition order, standard AC-PC orientation).

### Statistical analysis

Data were analyzed and visualized using Brain Voyager QX1.10 (Brain Innovation, Maastricht, Netherlands). Volumes were slice-time corrected and realigned to the first volume, normalized into standard Talairach space with parameters obtained from the co-registered high-resolution structural volumes, and smoothed with a Gaussian isotropic kernel (8 mm full width-half maximum). To remove low frequency drifts, data were high-pass filtered (3 cycles, 3 sine waves within the extent of the data) and *z*-transformed in each voxel separately. The BOLD response function was modeled by convolving the trial onsets with a standard hemodynamic response function. Motion-correction parameters were included in the model as confounding covariates at the first level. Effects of interest were estimated in each subject separately and brought to the second level to account for a random effect of subjects. Data were analyzed using a 2 (participant group)×2 (time)×2 (sentence) factorial design. The main effect of interest of the study was the interaction between the main effects of group (patients vs. healthy control subjects) and time (baseline month 1 vs. endpoint month 15) on the activation detected by the contrast personally relevant vs. neutral. To identify regions associated with changes we performed a whole-brain estimation of the model voxel by voxel, considering a priori clusters larger than 20 voxels. Because effect sizes obtained in an interaction may be expected to be small, we relied on the results of Fu et al. [22] to support inference and considered the extent to which our result confirmed findings of this previous study. The region of interest emerging from this study, and from the comparison with the existing literature on depression and its therapy, were the amygdala-anterior hippocampus region, and prefrontal areas as markers of emotion regulation processes [22], [31], [33], and more specifically on the medial surface [22], given the inconsistency of reports on the lateral surface/dorsolateral prefrontal cortex [45]. We report on additional findings with explorative intent. Anatomical identification of foci relied on publicly available empirical cytoarchitectonic maps [46]–[47] where these maps exist (amygdala and hippocampus). In the regressions of clinical response on signal changes, confounding by initial conditions was avoided by including baseline scores (BDI or GSI) and signal as covariates in the model (as in ref. [31]).

## Results

### Clinical Response

We first looked at changes in depressive symptoms to ensure that patients had responded to therapy. BDI scores refer to the level of depressive symptoms, whereas GSI scores reflect general symptomatic distress. At baseline, BDI score mean was 24.4 (standard deviation 9.5, range 10 to 40). BDI scores from 10 to 18 suggest mild to moderate depression; from 19 to 29 moderate to severe depression [48]. At endpoint (i.e. at the time of the second scan), the mean BDI score was 12.9 (standard deviation 8.2, range 2.5 to 35). Mean reduction in scores was 11.47 (paired *t*_15_ = 4.99, *p*<0.001). Mean GSI score at baseline was 1.35 (standard deviation 0.57, range 0.19 to 2.52), at endpoint 0.69 (standard deviation 0.36, range 0.16 to 1.41). Mean GSI score reduction was 0.66 (paired *t*_15_ = 5.99, *p*<0.001). Clinically, five patients still fulfilled diagnostic criteria for major depressive disorder at endpoint. All patients were planning at the end of the study to continue psychotherapy.

At baseline, controls showed scores in the healthy range in both GSI (mean score 0.18, standard deviation 0.13) and BDI (mean score 2.17, standard deviation 2.48). GSI and BDI scores did not change in controls. At endpoint, mean GSI score was 0.13 (standard deviation 0.11) and mean BDI score was 1.94 (standard deviation 2.36; GSI: paired *t*_16_ = 2.11, *p* = 0.052; BDI: paired *t*_17_ = 1.07, *p* = 0.299).

### Neuroimaging data

**Figure 2:**
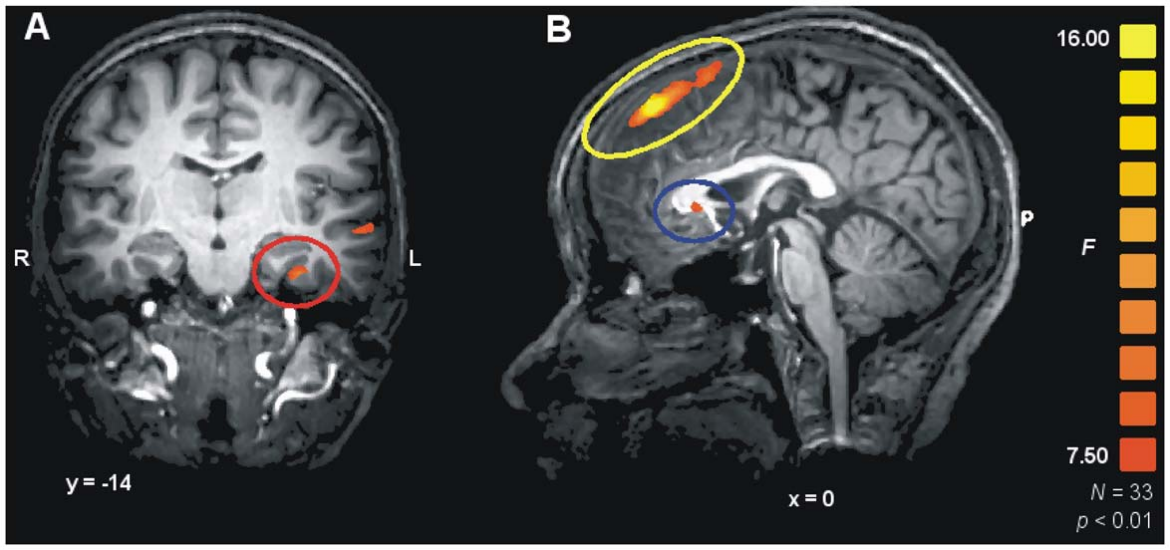
Neuroimaging results. Statistical maps of the interaction between contrast ‘relevant’ vs. ‘neutral’, group, and time, overlaid on a template T1-weighted brain in Talairach space. A: frontal slice showing the interaction in the amygdala/anterior hippocampus (red circle), and B: in the subgenual cingulus (blue) and in the anteromedial prefrontal cortex (yellow circle). For display purposes, the *F* map of the interaction was thresholded at *p* = 0.01, uncorrected.

The main effect of interest of the study was given by the interaction between group (patients and controls) and time (pre vs. post) for the contrast relevant vs. irrelevant, as this interaction directly detects changes intervening during therapy that affected patients but not controls in the appraisal of personalized material. In this interaction an effect in the left amygdala was detected ([46], Talairach coordinates *x*, *y*, *z*: −33, −11, −24, *F*_1,32_ = 9.00, *p* = 0.005), extending laterally into the anterior hippocampus where it reached its peak in Brodmann area (BA) 36 (*x*, *y*, *z*: −33, −14, −25, *F*_1,32_ = 12.11, *p* = 0.001, Figure 2A, red circle), and towards the middle temporal gyrus. On the right, the interaction failed to reach significance (*x*, *y*, *z*: 24, −10, −27, *F*_1,32_ = 3.24, *p* = 0.08), but this was consistent with the general left-lateralized pattern of activation elicited by the task. Post-hoc analysis revealed this effect to be due to patients showing more activity than control subjects at baseline (*x*, *y*, *z*: −33, −14, −25, *t*_32_ = 2.81, *p* = 0.008), which equalized or partially reversed at endpoint (*t*_32_ = −2.41, *p* = 0.01).

An interaction effect was also present in the ventral anterior cingulate cortex (vACC, *x*, *y*, *z*: 0, 23, 4, BA25, *F*_1,32_ = 6.91, *p* = 0.013, Figure 2B, blue circle). Post-hoc analysis identified this interaction as being mainly due to patients activating less when exposed to self-referential material at endpoint than controls (*t*_32_ = −2.1, *p* = 0.02), while at baseline the relation was partially inverted, with patients activating more than controls (*t*_32_ = 1.74, *p* = 0.05). A fairly large area of interaction involved the medial prefrontal cortex in a much more dorsal position (*x*, *y*, *z*: 3, 44, 49, BA8-9, *F*_1,32_ = 13.47, *p*<0.001, Figure 2B, yellow circle), which extended onto the lateral aspect in the superior frontal gyrus and posteriorly reached the middle frontal gyrus. This interaction was due to cortical activation in patients at baseline relative to controls (*t*_32_ = 3.00, *p* = 0.003) that equalized at endpoint (*t*_32_ = −1.6, n.s.). No other clusters of interaction in either direction were present with a size larger than 150 mm^3^ (about 20 voxels), even when selected at the liberal threshold *p*<0.05, uncorrected.

### Correlation of clinical response and cerebral activity

**Figure 3:**
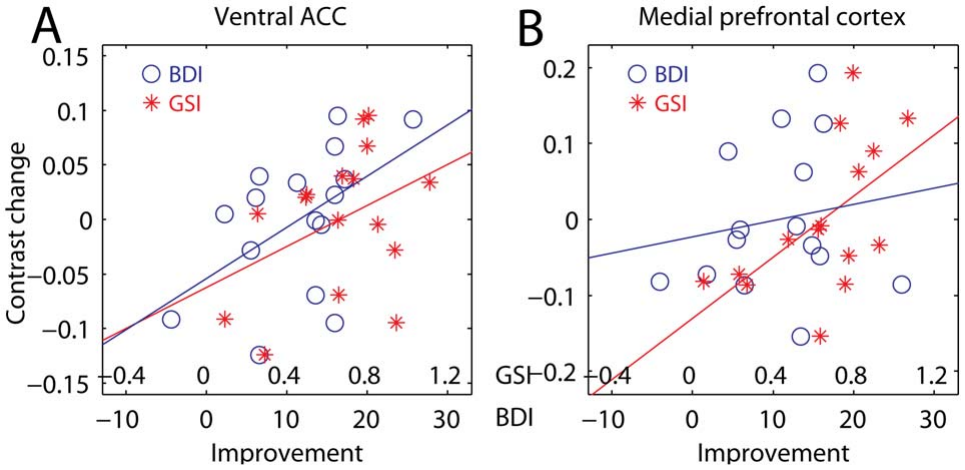
Association between neuroimaging data and improvement. The regression of the interaction effect on BDI (blue circles) and GSI (red stars) improvement is shown for the subgenual cingulus (A) and the medial prefrontal cortex cluster (B).

To verify that the areas detected by the interaction were related with changes in depressive symptoms, we regressed changes in the signal of the contrast personally relevant vs. neutral on the improvement in BDI scores, adjusting for initial levels to avoid confounding for initial severity. In the hippocampal/amygdalar cluster, the association was only at trend level (*t*_12_ = 1.29, *p* = 0.11), accounting for only 12% of the change in the signal from baseline to endpoint. A stronger association was found in the ventral ACC (*t*_12_ = 2.19, *p* = 0.02, explained variance 29%, Figure 3A, blue), which did not change when age and sex were included as covariates in the analysis (28%). In the medial prefrontal cortex the association between the interaction effect and clinical response was not significant (*t*_14_ = 0.92, *p* = 0.19).

Long-term psychodynamic psychotherapy is indicated for the treatment of chronic depression [9], and may be expected to address a wide range of issues that may complicate the depressive illness and contribute to chronicity. For this reason, we extended our analysis of the association of changes with response to the Global Severity Index (GSI [42]), a more general index of psychic well being than BDI. GSI and BDI scores were moderately associated at baseline (*r* = 0.32, *z* = 1.80, *p* = 0.04, 11% explained variance), but strongly correlated at endpoint (*r* = 0.87, *z* = 7.04, *p*<0.001, 76% of explained variance), suggesting effective treatment of initial non-depressive symptoms captured by the GSI score (interaction of the BDI/GSI association with time, *t*_28_ = 2.36, *p* = 0.01).

As in the previous analysis with BDI scores, there was no significant association between GSI improvement and the change in the contrast signal in the hippocampal/amygdalar cluster (*t*_12_ = 1.08, n.s.). However, a significant association was found in the ventral ACC cluster (*t*_12_ = 1.77, *p* = 0.05, explained variance 21%), which was not changed by including sex and age as covariates (20%). As one can see in Figure 3A, the association with GSI improvement (in red) did not differ much from that of BDI (in blue). Also in the medial prefrontal cortex there was an association between the change in the contrast signal and clinical response measured by GSI (*t*_12_ = 2.05, *p* = 0.03, explained variance 26%), which remained after including age and sex as confounding covariates (28%). As shown in Figure 3B, changes in the contrast signal were more strongly associated with the general improvement measured by GSI (in red) than with recovery from depression as reflected by the BDI scores (in blue).

## Discussion

When exposed to personally attachment-relevant material, patients undergoing long-term psychodynamic psychotherapy showed changes in brain activation that were not observed in a sample of control participants. The significant association of the changes in the subgenual cingular and medial prefrontal cortex with symptom improvement supported the hypothesis of their relevance to the changes intervened during therapy.

Among the areas involved by these changes, the anterior hippocampus/amygdalar complex has been shown to be implicated in the detection of emotional stimuli [49]–[52] and displays enhanced reactivity in depression [4]–[8], [11]–[12], [16] and anxiety [53]–[56]. The hippocampal/amygdalar correlate of change of the present study fell within 5–10 mm of the anterior hippocampal area associated with therapy change by Fu et al. [22] using cognitive behavioral therapy. Changes in the ventral ACC were located in the subgenual area reported by previous studies [36]–[37], [57]–[59] that provided converging evidence of its critical involvement in both mood dysregulation and its resolution [60]–[61]. Changes over time in both areas consisted of a reduction in the reactivity of these areas in response to personally relevant material in patients. This pattern was not observed in controls.

A third area, located anteriorly and superiorly in the medial prefrontal cortex, was found in the present study to change from baseline to endpoint in patients and not in controls. This area has been associated with voluntary emotion regulation [19], [62]–[63]. In the present study, this area displayed increased activation when exposed at baseline to personally relevant material in patients, and equalized at endpoint. Furthermore, changes in this area were associated with changes in general symptom severity rather than with changes in depressiveness specifically. This finding is consistent with research showing this area's general role in emotion regulation and control processes.

It is interesting that the prefrontal areas in which changes were detected in the present study correspond to those associated with emotion regulation [19], [62]–[63]. Studies of emotional appraisal and regulation have associated intentional avoidant and emotion suppression styles with increased psychiatric morbidity and vulnerability to depression [19]. Because the material in the present study was carefully chosen to represent personally relevant attachment themes, we consider the associated emotional appraisal to reflect the style of affect regulation.

There were several aspects of this study that are worth commenting. Among its strengths was the extension of neuroimaging approaches to the investigation of changes during therapy in conditions not investigated in previous studies: the recurrent type of the depression from which patients suffered, the length of therapy, and the psychodynamic nature of treatment. There were also several limitations. First, while the personalized design may have allowed an increase in validity and sensitivity of the stimuli used in the scanner [11], [30]–[32], it also introduces a possible confound due to the existence of systematic differences in the material produced by patients and controls. However, the absence of differences in the rating of the emotional arousal of these personalized sentences between patients and controls makes the existence of this confound less likely. Second, the study lacked a natural course control group composed by depressed patients on a waiting list. Given the length of time during which the study was conducted, keeping recurrent depressive patients on a waiting list would have been unethical.

The pattern of changes in prefrontal areas found in the present study may be specifically associated with mechanisms of emotional appraisal and control, suggesting reduced recourse to styles characterized by suppression and avoidance after long-term therapy. This interpretation outlines a possible mechanism for the understanding of emotional appraisal and regulation in the psychodynamic psychotherapy of depression. The relevance of these finding for future studies rests in the possibility of documenting specific mechanisms of action of depression therapy by systematically collating results from different studies and comparing different psychotherapeutic approaches, such as psychodynamic, behavioral, interpersonal, and psychopharmacological. This would be a first step towards monitoring progress of therapy in individual patients.
